# Fly Ash Porous Material using Geopolymerization Process for High Temperature Exposure

**DOI:** 10.3390/ijms13044388

**Published:** 2012-04-10

**Authors:** Mohd Mustafa Al Bakri Abdullah, Liyana Jamaludin, Kamarudin Hussin, Mohamed Bnhussain, Che Mohd Ruzaidi Ghazali, Mohd Izzat Ahmad

**Affiliations:** 1 School of Material Engineering, University Malaysia Perlis (UniMAP), P.O. Box 77, D/A Pejabat Pos Besar, Kangar, Perlis 01000, Malaysia; E-Mails: liyanajamaludin@unimap.edu.my (L.J.); vc@unimap.edu.my (K.H.); ruzaidi@unimap.edu.my (C.M.R.G.); ifan_7818@yahoo.com (M.I.A.); 2 King Abdul Aziz City Science & Technology (KACST), P.O. Box 6086, Riyadh 11442, Kingdom of Saudi Arabia; E-Mail: bnhusain@kacst.edu.sa

**Keywords:** geopolymer, pozzolanic material, thermal analysis

## Abstract

This paper presents the results of a study on the effect of temperature on geopolymers manufactured using pozzolanic materials (fly ash). In this paper, we report on our investigation of the performance of porous geopolymers made with fly ash after exposure to temperatures from 600 °C up to 1000 °C. The research methodology consisted of pozzolanic materials (fly ash) synthesized with a mixture of sodium hydroxide and sodium silicate solution as an alkaline activator. Foaming agent solution was added to geopolymer paste. The geopolymer paste samples were cured at 60 °C for one day and the geopolymers samples were sintered from 600 °C to 1000 °C to evaluate strength loss due to thermal damage. We also studied their phase formation and microstructure. The heated geopolymers samples were tested by compressive strength after three days. The results showed that the porous geopolymers exhibited strength increases after temperature exposure.

## 1. Introduction

In 1978, Davidovits introduced the word “geopolymer” to describe an alternative cementitious material which has ceramic-like properties [1]. Geopolymer technology has the potential to reduce emissions by 80% because high temperature calcining is not required [2]. It also exhibits ceramic-like properties with good resistance to fire at elevated temperature. Geopolymer are amorphous to semi crystalline equivalent of certain zeolitic materials with excellent properties such as high fire and erosion resistances and high strength materials [3]. Materials that use fewer natural resources, require less energy, and generate less CO_2_ are referred to as green materials. Fly ash refers to the inorganic, incombustible matter present in coal that is fused into a glassy, amorphous structure during the combustion process [4]. Geopolymer is a man-made material with many exceptional properties including impressive fire resistance and the capacity to encapsulate hazardous waste [5].

The alkaline liquid could be used to react with the silicon (Si) and the aluminum (Al) in a source material of natural minerals or in by-product materials such as fly ash and rice husk to produce binders [6]. Fly ash geopolymer does not require high temperature processing. Fly ash-based geopolymer with 12 M NaOH concentration shows excellent result with high compressive strength (94.59 MPa) for the 7th day of testing [3]. Palomo *et al*. (1999) reported that an activator with a 12 M of NaOH concentration leads to better results than an 18 M of NaOH concentration. In addition, the researchers used a ratio of fly ash to alkaline activator in the range of 2.5 to 3.3 to achieve optimum parameters with better strength [3]. Samples cured at 70 °C provided the concrete with good strength and workability properties [7]. Some researchers have described the alkali activation of fly ash (AAFA) as a physical–chemical process in which the powdery solid is mixed with a concentrated alkali solution in a suitable proportion to produce a workable and mouldable paste, which is stored at mild temperatures (<100 °C) for a short period of time to produce a material with good binding properties [8].

High temperature performance of geopolymers are investigated and presented in this paper with a view to produce an alternative refractory material. Cement is produced by heating a powdered mixture of limestone, clay, ferrous materials, and siliceous materials to temperature of 1500 °C [1]. Geopolymer can be produced by combining pozzolanic compound or aluminosilicate source material with highly alkaline solution [5]. Their physical behavior is similar to that of OPC and has been considered as a possible improvement on cement in respect of compressive strength, resistance to fire, heat and acidity, and as a medium for the encapsulation of hazardous or low/intermediate level radioactive waste [9–12]. In the present work we briefly investigated a geopolymer which is suitable for high temperature application.

## 2. Results and Discussion

### 2.1. Physical Behavior of Porous Geopolymer after Sintering

Physical observations showed the decolorization of all specimens after sintering. The dimensional change did not exhibit after sintering at high temperature. Observations showed few cracks after exposure to elevated temperatures. The average mass reduction compared with geopolymer before sintering for different mass ratios and temperature are summarized in Table 1.

### 2.2. Compressive Strength

**Figure 1:**
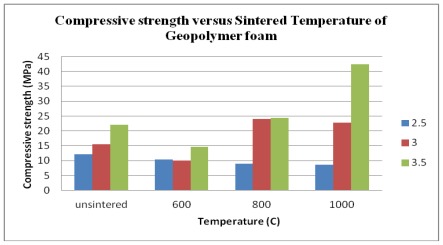
Effect of unsintered and sintered temperature of porous geopolymer on compressive strength.

The compressive strength of porous geopolymer samples were measured using mechanical testing with Automatic Max (Instron, 5569 USA). The strength of samples was tested three days after casting. Physical observations showed the decolorization of all specimens. Figure 1 represents the compressive strength development of porous geopolymer samples before and after temperature exposure with various Na_2_SiO_3_/NaOH ratio results ranging from 2.5 to 3.5. The compressive strength of geopolymer significantly improved with increasing sintering temperature and as the ratio increased. An increase in Na_2_SiO_3_/NaOH ratio results in an increase of Na content in the mixtures which in turn produce more stable strength properties [13]. Rapid strength development occurs within geopolymers with higher concentrations of NaOH [14]. As presented in Table 2, the highest compressive strength, 42.40 MPa, was achieved after three days with the 3.5 ratio of Na_2_SiO_3_/NaOH and highest sintering temperature of 1000 °C. Geopolymer samples withstand a maximum application temperature of approximately 1000 °C [15].

### 2.3. Scanning Electron Microscopy Analysis

**Figure 2:**
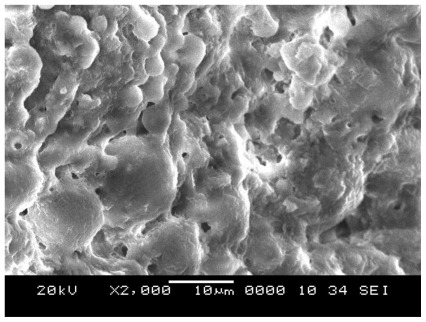
Porous geopolymer microstructure before temperature exposure.

The microstructure of porous geopolymer in Figure 2 shows the formation of porous heterogeneous matrix which does not exist in the original fly ash. It can be seen that porous geopolymers with lower ratio have more open porosity and the open porosities of porous geopolymers decrease with increasing heat treatment temperatures. The increase in porosity is attributed to sintering possibility by assistance from a liquid phase.

**Figure 3:**
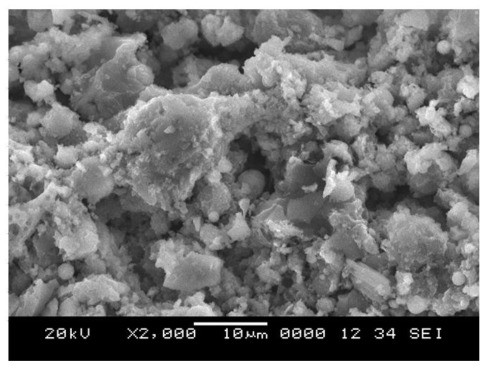
Porous geopolymer after 1000 °C temperature exposure.

**Figure 4:**
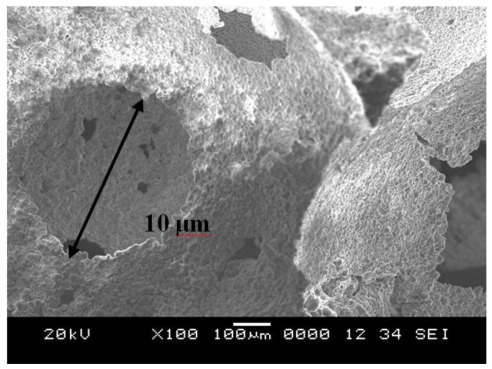
Hollow cavities of geopolymer porous sample.

The SEM image for the porous geopolymer heated to 1000 °C shows a microstructure that appears to contain relatively less unreacted fly ash microspheres proportions. Porous geopolymer after temperature exposure is shown in Figure 3. Figure 4 shows hollow cavities due to spaces left behind by dissolved fly ash particles. The pore sizes in the geopolymer after temperature exposure were 10 μm–20 μm. Porosity is caused by different developments of microstructure skeletons in the porous geopolymer. The solid-to-liquid ratio affects the volume of voids and porosity in the geopolymer which directly influences the strength of geopolymer.

### 2.4. Water Absorption and Porosity

The water absorption of geopolymer samples was determined with different water/solids ratios. Water absorption can be used to represent an open porosity of geopolymer. The measurement is taken by calculating the difference in specimen weight under over-dried and fully saturated conditions. The percentage of water absorption for all geopolymer samples varied in the range 2.56 to 4.49% at three days of the geopolymer age. The water absorption of fly ash geopolymer normally varies between 3 and 5% [16]. When high alkaline solution is added to the mixture, the water absorption tends to increase. A mix with a high amount of alkaline activator solution will produce a more porous geopolymer gel. A high amount of sodium silicate ratio in a mixture was found to produce geopolymer concrete with large pore sizes [17]. This explains the tendency of a mixture with high alkaline content to have higher porosity than mixes with low alkaline ratios.

## 3. Experimental Setup

### 3.1. Materials

Fly ash obtained from Manjung power station in Lumut, Perak, Malaysia was used as base materials to produce the geopolymers sample.

Sodium silicate (Na_2_SiO_3_) mixed with sodium hydroxide (NaOH) used as an alkaline activator for this research. NaOH in pellet form with 97% purity (Vijaya Rangan, 2008) and Na_2_SiO_3_ consists of Na_2_O = 9.4%, SiO_2_ = 30.1% and H_2_O = 60.5%, with mass ratio SiO_2_/Na_2_O = 3.20–3.30). Foaming agents, including spherical Al powder and hydrogen peroxide, were added to the geopolymer paste to control porosity into the material and to shorten the diffusion distance for entrapped water to leave the samples.

### 3.2. Mixture Compositions

Geopolymer materials were prepared by mixing alumino-silicate with the alkaline activator solution. The alkaline activator solutions were prepared by the dissolution of sodium hydroxide in one liter of distilled water in a volumetric flask to obtain a 12 M concentration. Alkaline activator, which consisted of the combination of NaOH and Na_2_SiO_3_, was prepared just before it was to be mixed with the fly ash. The sodium silicate was added to enhance the process of geopolymerization [18]. The ratio of pozzolanic materials/alkaline activator and Na_2_SiO_3_/NaOH used was 2.5, 3.0 and 3.5 for all mixtures. This particular ratio was used based on the work of Hardjito *et al.* (2008) in which it was stated that this ratio produced the highest compressive strength [19]. Table 3 shows a mixture with proportions of geopolymer paste with different ratios of Na_2_SiO_3_/NaOH.

### 3.3. Samples Preparation

The alkaline solution was added to the geopolymeric precursor (fly ash) and mixed for 5 min to obtain a homogeneous mixture. Foaming agent solution was added to geopolymer paste to get porous geopolymer. The geopolymer mixture formed a slurry paste and was poured into a steel sample mould (50 mm × 50 mm × 50 mm) which was then allowed to stand at room temperature for a few hours.

### 3.4. Curing Regime and High Temperature Exposure

Geopolymer paste was cured in the oven for one day at constant temperature of 60 °C [7]. At the end of curing regime, the samples were removed from their molds and allowed to cool before initial qualitative observations were recorded. To study the effect of heating on the microstructure and loss of water, the cured porous geopolymer were heated at 600 °C, 800 °C, and 1000 °C for three hours in box furnace with heating and cooling rates 5 °C/min [20]. The unexposed samples were left undisturbed at ambient temperature.

### 3.5. Compressive Test

The compressive strength of geopolymer samples were measured according to BS 1881-116:1983 using mechanical testing with Automatic Max (Instron, 5569 USA) in order to obtain the ultimate strength of geopolymer. The samples were compressed with 50 kN load and with loading rate of 5 mm/min.

### 3.6. Microstructure Analysis Test

Scanning electron microscopy (SEM) was performed using SEM JSM-6460 LA Jeol Japan in School of Materials Engineering, University Malaysia Perlis (UniMAP) to analyze the microstructure of the fly ash and porous geopolymer samples. The test was conducted using secondary and backscattered electron detectors. SEM analysis was done at the accelerating voltage of 20 kV. Samples of fly ash were prepared in powder form and the other porous geopolymer samples were placed on the sample holders supported by carbon tape.

## 4. Conclusions

In conclusion, the fly ash porous geopolymer displayed increase in strength after temperature exposure of 1000 °C. This is attributed to the increase in a combination of polymerization reaction and sintering at high temperature. The fly ash-to-activator ratio can be an important parameter regarding strength and sintering temperature of the porous geopolymer. Fly ash porous geopolymer with a ratio 3.5 of Na_2_SiO_3_/NaOH shows excellent results with high compressive strength (42.4 MPa) after 3 days of testing, even after high temperature exposure.

## Figures and Tables

**Table 1:** Percentage of mass reduction of sintered porous geopolymer samples.

Ratio Na_2_SiO_3_/NaOH	Mass reduction (%)		

	600 °C	800 °C	1000 °C
2.5	17.32	19.45	22.36
3.0	17.80	19.50	22.65
3.5	17.95	19.70	27.35

**Table 2:** Geopolymer samples strength results.

Ratio Na2SiO3/NaOH	Compressive strength after 3 days (Mpa)			

	unsintered	600 °C	800 °C	1000 °C
2.5	12.12	10.41	8.90	8.63
3.0	15.56	10.04	24.00	22.83
3.5	21.99	14.63	24.33	42.40

**Table 3:** Mixture proportions of geopolymer paste.

Mixture of geopolymer	Mass ratios (2.5)	Mass ratios (3.0)	Mass ratios (3.5)
Ratio of fly ash/alkaline activator	2.5	3.0	3.5
Ratio Na_2_SiO_3_/NaOH	2.5	3.0	3.5
Mass of fly ash (g)	1210	1270	1310
Mass of NaOH (g)	137.8	105.5	83.3
Mass of Na_2_SiO_3_ (g)	344.4	316.4	291.7
